# Assessment of grief-related rumination: validation of the German version of the Utrecht Grief Rumination Scale (UGRS)

**DOI:** 10.1186/s12888-018-1630-1

**Published:** 2018-02-09

**Authors:** Bettina K. Doering, Antonia Barke, Thilo Friehs, Maarten C. Eisma

**Affiliations:** 1 Clinical Psychology & Psychotherapy, Department of Psychology, Philipps-University Marburg, Marburg, Germany; 2 Department of Clinical Psychology and Experimental Psychopathology, University of Groningen, Groningen, The Netherlands

**Keywords:** Rumination, Repetitive thinking, Bereavement, Complicated grief, Assessment, Validation

## Abstract

**Background:**

Bereavement can result in severe mental health problems, including persistent, severe and disabling grief symptoms, termed complicated grief. Grief rumination (i.e., repetitive thought about the causes and consequences of the loss) is a malleable cognitive risk-factor in adjustment to bereavement. The Utrecht Grief Rumination Scale (UGRS) was recently developed to assess grief rumination. The present study aimed to develop and validate a German version of the UGRS.

**Methods:**

An online survey including measures of demographic and loss-related variables, grief rumination (UGRS), depressive rumination (brooding and reflection), and symptoms of depression, anxiety, and complicated grief, was administered online among 159 persons (87% women) who had lost a first-degree relative in the past three years. UGRS item analyses, a confirmatory factor analysis and associations of grief rumination with brooding, reflection and symptom levels were performed.

**Results:**

The internal consistency of the UGRS was good. The confirmatory factor analysis obtained a good fit for a model with five correlated grief rumination subscales. The UGRS contributed uniquely to the prediction of complicated grief symptoms even when controlling for symptoms of anxiety and depression, brooding, reflection, and demographic and loss-related variables. Discriminant validity of the UGRS was demonstrated by the fact that higher UGRS scores were found in participants with a higher likelihood of receiving a diagnosis of complicated grief (*d* > 1.60).

**Conclusion:**

The translated UGRS showed very good psychometric properties and the correlations with maladaptive ruminative styles and complicated grief symptoms demonstrated the clinical relevance of grief rumination. Limitations concerning generalisability of the results are discussed.

**Electronic supplementary material:**

The online version of this article (10.1186/s12888-018-1630-1) contains supplementary material, which is available to authorized users.

## Background

Bereavement is a universal human experience. While most people react to a loss with intense pain and may even develop an increased risk of mental or physical health problems [1], a majority adjust successfully to this stressful life-event without professional help. A minority of bereaved people, however, suffer from persistent grief symptoms of clinical relevance that are accompanied by functional impairment. Both new diagnostic classification systems of mental health conditions acknowledge the syndrome. The Diagnostic and Statistical Manual for Mental Disorders in its 5th edition (DSM 5) classifies it as ‘Persistent Complex Bereavement Disorder’ (PCBD) and considers it a condition for further study [2]. The International Classification of Diseases in its 11th edition (ICD-11) will probably include ‘Prolonged Grief Disorder’ (PGD) as a stress-related disorder [3, 4]. Concerning symptom duration, ICD 11 may allow a diagnosis as early as six months after the loss occurred, whereas DSM 5 requires symptoms to last twelve months. A recent meta-analysis estimates the pooled prevalence of PGD after bereavement to be 9.8% [5]. The present article will use the well-established term ‘complicated grief’ to refer to clinically relevant grief symptomatology, since the present assessment of grief symptoms follows the Inventory of Complicated Grief [6]. This also allows for drawing on existing research of grief rumination and bereavement outcome.

Given the severe distress that may be experienced after bereavement, a thorough understanding of the malleable factors that may contribute to the development and persistence of mental health problems is important. Thought processes are both malleable (for a review: [7]) and are assumed to play an important role in the potential transition from ‘normal’ to complicated grief and its maintenance [8]. Special attention has been paid to trans-diagnostic thought processes such as repetitive thinking, i.e. the ‘process of thinking attentively, repetitively or frequently about one’s self and one’s world’ ([9], p. 909). Repetitive thinking about the deceased, the loss and its circumstances and consequences seems inherent to the acute grieving process [10]. Some forms of repetitive thinking, however, such as rumination, have been associated with poor bereavement outcome, both concurrently and prospectively (cf. [11–13]).

Two types of rumination have been studied in some detail in adjustment to loss. The first investigations in this area were conducted in the mid-nineties. They focused on clarifying the role of depressive rumination after bereavement. Depressive rumination was defined as repetitively and passively focusing on depressive symptoms and on their possible causes and consequences [11]. A frequently used theory to understand the effects of depressive rumination on psychopathological symptoms is the Response Styles Theory (RST). The RST proposes that depressive rumination fuels depression by increasing the accessibility of negative thought content, impairing instrumental behaviour and problem solving, and driving away social support [11, 14]. In an attempt to differentiate adaptive from maladaptive forms of depressive rumination (cf. [15]) and to minimise the content overlap of the assessment of rumination with that of symptoms of depression, two sub-facets of depressive rumination were introduced, namely brooding and reflection [16]. ‘Brooding’ implies a passive comparison of the aversive current situation with some unachieved standard, and ‘reflection’ indicates actively focusing inward to engage in cognitive problem solving in order to overcome depressive symptoms. Longitudinally, brooding has been associated with more depressive symptoms, whereas reflection seems to be associated with less depressive symptoms [16]. Concerning adaptation to bereavement, RST conceptualises rumination as a confrontation strategy as it entails thinking repeatedly about post-loss emotions. Previous research indicates that all three constructs (i.e. depressive rumination, brooding, and reflection) are associated with psychopathological symptoms after bereavement [17–23]. However, it also suggests that another type of rumination, namely grief rumination, is potentially more predictive of mental health problems in adjustment to bereavement, consistently explaining more variance in post-loss symptoms of depression, posttraumatic stress and complicated grief concurrently and longitudinally ([20, 24, 25]; for a review: [13]).

In contrast to depressive rumination, grief rumination is not limited to analysing feelings of depression, as negative post-loss emotions are not restricted to sadness or helplessness but may also entail many other emotions including yearning, anger or irritability [1]. Thus, rumination after loss likely focuses on a wider array of loss-related feelings [19]. Additionally, typical topics of rumination will differ in grief and depression. Similarly to rumination after traumatic events [26, 27], grief rumination may focus strongly on reconciling the event with previously held beliefs about the meaningfulness or fairness of the world (i.e., thinking about why the event happened and the injustice of the loss), and counterfactual thinking (i.e., thinking about possible courses of action that might have prevented the event’s occurrence).

A model that is currently often used to understand the negative consequences of grief rumination is the Rumination as Avoidance Hypothesis (RAH [10]). The RAH conceptualises rumination as an avoidance strategy because when ruminating about, for example, alternative outcomes of the situation (counterfactual thinking), one may avoid confronting the reality and permanence of the loss. Rumination would thus impede acceptance of the loss and hinder its contextualisation within existing autobiographical knowledge [8]. Previous research suggests that experiential avoidance and thought suppression longitudinally mediate the relationship between grief rumination and symptoms of complicated grief [25]. Experimental approaches have also corroborated the link between grief rumination and avoidance [28, 29]. Additionally, grief rumination has been investigated in another longitudinal study of recently bereaved participants [20]. In this sample, while simultaneously controlling for baseline symptom levels and other loss-related variables, grief rumination was a stronger predictor of later symptom levels of grief than was depressive rumination. This analysis also provided the first evidence of a distinction between adaptive and possibly maladaptive facets of grief rumination. Rumination about emotional reactions to the loss was regarded as potentially adaptive, since it was longitudinally associated with lower symptom levels. Rumination about the injustice of the loss was considered potentially maladaptive, since it was longitudinally associated with higher symptom levels.

Given grief rumination’s potential theoretical and clinical relevance, the Utrecht Grief Rumination Scale (UGRS) was recently developed to specifically assess grief rumination [19]. The UGRS is based on theories of depressive rumination [16, 30], trauma-related rumination [26], and grief-relevant rumination [8]. It captures five typical themes of post-loss rumination: (1) personal emotional reactions to the loss, (2) injustice of the death, (3) counterfactual thoughts about the circumstances of the death, (4) meaning and consequences of the loss, and (5) the reactions of others to the loss. It was originally published in Dutch [24]; an English version has been developed and its cross-cultural equivalence confirmed [19]. In confirmatory factor analyses of the data of the Dutch and British samples, a single-level factor structure with five correlated factors provided the best model fit, even though a hierarchical model with a second-order factor performed almost equally as well [19]. In English and Dutch samples, the UGRS has demonstrated very good psychometric properties. It showed excellent internal consistency (α = .90) and, as a first indication of its validity, the UGRS contributed to the prediction of depression, posttraumatic stress and complicated grief over and above demographic and loss-related variables and other measures of rumination [19, 24].

Clearly, more international research is needed to distinguish potentially adaptive and maladaptive facets of rumination at different time points in the grieving process. We also need to elucidate the pathways via which rumination contributes to the development and maintenance of mental health problems and, specifically, complicated grief. As prerequisite to this long-term goal, the present study aimed to develop a German version of the UGRS, to investigate its psychometric properties (e.g. reliability, item-correlations, factor structure), and to test its concurrent and discriminant validity by examining associations between the UGRS, reflection and brooding, and symptoms of anxiety, depression and complicated grief.

## Methods

### Participants and procedures

Ethical approval was obtained from the Ethics Committee of the Department of Psychology of the Philipps University Marburg (Germany) and invitations to participate were posted on grief-related websites (e.g., peer support websites). Recruitment lasted from August to October 2016. People accepting the invitation followed a link to an online survey platform that offered information about the aims of the study, confidentiality and study eligibility criteria; after providing written informed consent, they could participate in the study. As an incentive, participants had the chance of winning one out of two 50€-vouchers for an online store. Inclusion criteria were age over 17 years and having lost a first-degree relative or partner within the last three years. Exclusion criteria were feeling too distressed by grief to answer loss-related questions or having experienced suicidal ideation in the last month. For the analysis in the present paper, participants who indicated that German was not their first language were excluded. A total of 195 participants gave informed consent; of these, 36 were excluded (German not first language, *n* = 5; premature termination of the survey, *n* = 28; systematic data pattern, *n* = 3). The final sample therefore consisted of 159 bereaved participants, who were mostly female (87%) and aged between 18 and 77 years (M = 47 years, SD = 12). Concerning time since loss, 23% indicated that the death had taken place less than six months ago, 21% that it had happened six to twelve months ago and most (55%) reported their loss dating back to between one and three years. Most participants (54%) had lost a partner/spouse, 37% had lost a child, 6% a parent and 3% a sibling. The mean Inventory of Complicated Grief (ICG) score in the present sample was 34.33 (±13.56); most participants (75%) demonstrated ICG scores higher than the established cut-off (> 25).

### Measures

#### Demographic and loss-related variables

In addition to the following questionnaires, participants were asked to provide demographic data (age in years; gender) and loss-related information. Loss-related variables comprised time since loss (less than 6 months; 6–12 months; 12 months to 3 years), relationship to the deceased (i.e., partner/spouse, parent, child, sibling), and cause of death (i.e., medical condition, natural death, accident, suicide, perinatal complication, unresolved cause, other).

#### Grief rumination

The English Utrecht Grief Rumination Scale (UGRS) [19] was translated into German following the guidelines by Beaton et al. [31]. The German version of the UGRS (UGRS-D) is provided as Additional file 1.

The UGRS consists of 15 items detailing various types of thought about the causes and consequences of the loss; participants indicate how frequently they have experienced each of these in the past month. Answers are rated on a five-point scale ranging from 1 (never) to 5 (very often). Item scores are summed to generate an overall grief rumination score. Furthermore, five subscales can be computed, consisting of three items each. ‘Reactions’ (items 6, 7, 13) measures how frequently participants analyse their emotional reactions to the loss (example item: ‘How often in the past month did you try to analyse your feelings about this loss precisely?’) The subscale ‘Injustice’ (items 5, 11, 12) captures thoughts about the injustice of the loss (example item: ‘How often in the past month did you wonder why this had to happen to you and not to someone else?’). ‘Counterfactuals’ (items 4, 8, 10) assesses counterfactual thinking about the events leading to the loss (example item: ‘How often in the past month did you analyse if you could have prevented the death?’). The subscale ‘Meaning’ (items 1, 2, 15) measures thoughts about the meaning and the consequences of the death (example item: ‘How often in the past month did you analyse what the personal meaning of the loss is for you?’). The subscale ‘Relationships’ (items 3, 9, 14) assesses thoughts related to reactions from the social environment (example item: ‘How often in the past month did you think about how you would like others to react to your loss?’). Eisma et al. [19] showed that the internal consistency was excellent for the UGRS (Cronbach’s α = .90), and reliability measures of the subscales were good to excellent (Reactions, Cronbach’s α = .84; Injustice, Cronbach’s α = .88; Counterfactuals, Cronbach’s α = .89; Meaning, Cronbach’s α = .84; Relationships, Cronbach’s α = .74).

#### Depressive rumination

The Response Style Questionnaire 10D (RSQ-10D) [32, 33] is the German version of the Response Style Questionnaire short form established by Treynor et al. [16]. It captures ruminative styles, i.e. the sub-facets ‘brooding’ and ‘reflection’ with proposed minimal content overlap with depressive symptoms. Both facets are each assessed by five items. Participants are asked to indicate how frequently they experience certain aspects of ruminative thinking on a four-point scale ranging from 1 (never) to 4 (almost always). An example item for brooding is: ‘Why do I always react this way?’. An example item for reflection is: ‘I write down what I am thinking and analyse it.’ The brooding and reflection scales have demonstrated adequate internal consistencies (Cronbach’s α in different samples: brooding: .60 ≤ α ≤ .75; reflection: .56 ≤ α ≤ .75) [32]. In this sample, Cronbach’s α for the subscales was .74 for brooding and .66 for reflection.

#### Symptoms of anxiety and depression

To measure symptoms of anxiety and depression, the Hospital Anxiety and Depression Scale (HADS) [34] was used in its validated German version (HADS-D) [35]. On subscales of seven items each, participants were asked how frequently, or to what extent, they experienced symptoms of anxiety or depression in the last week on a four-point scale with different verbal expressions for every item. An example item addressing ‘anxiety’ is: ‘I feel tense or wound up’. An (inverted) example item addressing ‘depression’ is: ‘I feel cheerful’. The subscales have good internal consistencies (anxiety: Cronbach’s α = .80; depression: Cronbach’s α = .81) and the scale has been validated in representative samples of the general population [35]. In this sample, Cronbach’s α for the subscales was .79 for anxiety and .88 for depression.

#### Complicated grief

The Inventory of Complicated Grief (ICG) [6] was used in its German version (ICG-D) [36]. It contains 19 items, which describe emotional, cognitive and behavioural states relevant to persistent disabling grief. Items are rated with regard to their current occurrence on a five-point Likert scale ranging from 0 (never) to 4 (always) and added to form a total score. The ICG-D has demonstrated excellent internal consistency (Cronbach’s α = .94) and good validity [36]. In this sample, Cronbach’s α was .90. A cut-off of 25 and above has been established as identifying more disabling states of grief [6] and can be used to identify potential ‘cases’ of complicated grief (e.g. [37, 38]). This cut-off (together with a time criterion, cf. Statistical analyses) was also used in the present study to identify ‘candidates’ for a diagnosis of complicated grief, bearing in mind that a self-report measure alone is not sufficient for establishing a diagnosis.

### Statistical analyses

In the UGRS, no missing data were observed; single missing items in other questionnaires were replaced according to the respective questionnaire’s instructions (i.e. replacement of single missing items by mean of the scale/subscale). Very few participants failed to provide answers to single items, namely: ICG *n* = 5; HADS *n* = 6; RSQ-D *n* = 6.

For the UGRS, standard item analyses were calculated to determine mean item scores and standard deviations, item difficulties, item-total correlations (with the item itself excluded from the total score) and estimations of internal consistency when the item was omitted. Mean inter-item correlations, mean item difficulties and internal consistency (standardised Cronbach’s α) of the UGRS and its subscales were computed. Confirmatory factor analyses with maximum likelihood estimation were calculated to test two models previously reported for the factorial structure of the English and Dutch versions [19]: The first model (model A) assumed five correlated factors (subscales) and the second model (model B) stipulated a single higher-order factor explaining the five subscales. Goodness of fit was assessed with the chi-square test, the root mean square error of approximation (RMSEA), the standardised root mean squared residual (SRMR), the comparative fit index (CFI) and the parsimony-adjusted comparative fit index (PCFI). The following are viewed as cut-off values indicating a good fit: χ/df ratio of ≥2, RMSEA< .05, SRMR< .06 and CFI ≥ .95 [39]. The Akaike Information Criterion (AIC) was computed as an index to compare the two models; while its absolute value is not informative, smaller AIC scores indicate a better model fit when comparing several models. Correlations of the UGRS with symptoms of complicated grief (ICG), anxiety and depression (HADS) and brooding and reflection as facets of rumination (RSQ-10D) were calculated; Bonferroni-corrected significance levels are reported to account for possible alpha error cumulation due to multiple comparisons. Reported correlation coefficients are Pearson coefficients; correlation coefficients were compared with Fisher’s z-test.

Various facets of validity were investigated. Convergent validity was assessed by inspecting zero-order correlations of UGRS and brooding. Divergent validity was assessed by calculating zero-order correlation of UGRS and reflection; z-tests compared the correlations of UGRS with brooding and reflection, respectively, since grief rumination should be less closely related to reflection. This prediction relies on the distinction between adaptive and maladaptive forms of repetitive thinking [15, 16, 40]. Multiple facets of the UGRS (i.e. grief rumination about injustice and social relationships) [20] have been identified as maladaptive forms of repetitive thinking; yet only one facet of the UGRS has been identified as potentially adaptive (i.e. rumination about reactions). Therefore UGRS total scores should generally demonstrate a closer association with maladaptive (i.e. brooding) than with adaptive forms of repetitive thinking (i.e. reflection). Concurrent validity was investigated by calculating zero-order correlations of UGRS and ICG and testing this correlation against other measures of psychopathology (anxiety and depression), using z-tests. To further examine concurrent validity, a hierarchical regression analysis examined the associations of the UGRS score with symptoms of complicated grief (criterion). Only participants whose loss had occurred more than six months ago were included, in order to exclude those acutely bereaved from this analysis. Using forced blockwise entry, demographic and loss-related variables were entered first. The second step additionally included symptoms of depression and anxiety (HADS), the third step entered brooding and reflection as more general rumination constructs. Lastly, grief-specific rumination (UGRS) was entered into the model. No outliers or influential cases were identified based on the leverage value criterion described by Stevens [41] or Cook’s distance values [42]. VIF and tolerance statistics indicated that no multicollinearity was present.

To investigate the discriminant validity of the UGRS, we compared potential candidates for a complicated grief diagnosis with non-candidates for such a diagnosis. A potential candidate for a complicated grief diagnosis was defined as a participant with an ICG score above the cut-off of 25 [6] who also fulfilled a criterion of how much time had passed since the loss. The time restriction was necessary to avoid misclassifications of acute grievers as candidates for a diagnosis of complicated grief. Currently, two possible time criteria are considered: more than six months (ICD-11) and more than twelve months (DSM 5). In order to account for both options, we used one independent t-test to compare participants above and below the ICG cut-off whose loss had occurred more than six months ago (ICD-11 time criterion) and a second t-test to compare participants above and below the ICG cut-off whose loss dated back more than twelve months (DSM 5 time criterion). If Levene’s test indicated that variances were unequal, the Welch test is reported (and the degrees of freedom were adjusted accordingly). Where appropriate, Cohen’s *d* is reported as a measure of effect size. The data analysis was carried out with IBM SPSS statistics 24; for the confirmatory factor analysis, the SPSS AMOS version 21.0.0 was used (IBM, Meadville, USA). Unless otherwise stated, the α level was set to *p* = .05.

## Results

### Item analysis

Item difficulties ranged between *p*_i_ = .37 (item 10) and *p*_i_ = .80 (item 2) with a mean difficulty of *p*_i_ = .54. The item-whole correlations of the individual items with the total score ranged from *r*_itc_ = .45 (item 10) to *r*_itc_ = .64 (item 9); the mean item-total correlation was *r*_itc_ = .57 and the mean inter-item correlation was *r* = .36 (see Table 1). The internal consistency of the whole scale was α = .89 and the consistency would not have benefitted from removing any item. Internal consistencies of the UGRS subscales were: UGRS _Meaning_ α = .86; UGRS _Relationships_ α = .82; UGRS _Counterfactuals_ α = .83; UGRS _Injustice_ α = .91; and UGRS _Reactions_ α = .72.

**Table 1:** Item analyses of the German UGRS

Item	Mean	SD	Item Difficulty *p*_i_	Corrected item-whole correlation *r*_itc_	Cronbach’s alpha if item is deleted
1	4.13	1.09	.78	.52	.89
2	4.21	1.10	.80	.56	.88
3	2.57	1.39	.39	.56	.88
4	2.80	1.53	.45	.58	.88
5	2.94	1.61	.49	.63	.88
6	3.55	1.25	.64	.51	.89
7	2.65	1.49	.41	.51	.89
8	3.21	1.49	.55	.62	.88
9	2.56	1.29	.39	.64	.88
10	2.50	1.56	.37	.45	.89
11	2.73	1.64	.43	.63	.88
12	3.30	1.53	.57	.62	.88
13	3.53	1.32	.63	.51	.89
14	2.97	1.28	.49	.51	.89
15	4.14	1.03	.78	.63	.88

### Confirmatory factor analysis

**Fig. 1:**
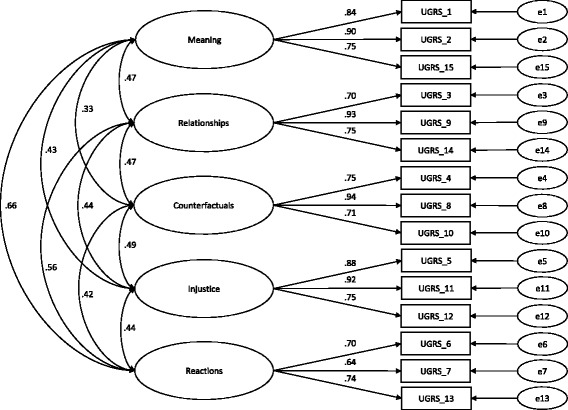
Confirmatory factor analysis of the UGRS (Model A). Path diagram for confirmatory factor analysis of the UGRS with five intercorrelated, first-order factors showing standardised path coefficients (Model A). Error terms are denoted with a small ‘e’. All path coefficients are significant at *p* < .001

**Fig. 2:**
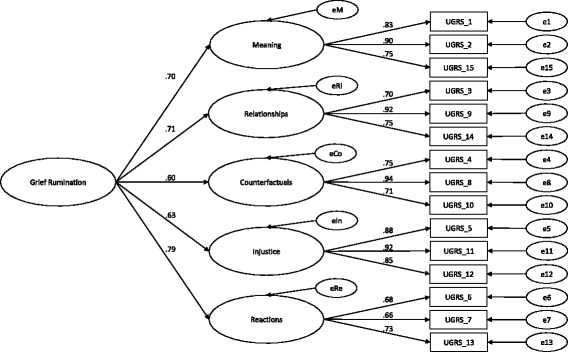
Confirmatory factor analysis of the UGRS (Model B). Path diagram for confirmatory factor analysis of the UGRS with the five first-order factors and the latent construct Grief Rumination as a higher-order factor showing standardised path coefficients (Model B). Error terms are denoted with a small ‘e’. All path coefficients are significant at *p* < .001. *M* Meaning subscale, *Rl* Relationships subscale, *Co* Counterfactuals subscale, *In* Injustice subscale, *Re* Reactions subscale

In order to test the previously established five-factor structure [19], a confirmatory factor analysis was carried out for the UGRS. First, we examined model A, containing just five correlated factors with the respective subscale items as indicators (Fig.1 for the path diagram). Secondly, we tested a more parsimonious model B, in which the five subscales are themselves the indicators for one higher-order factor, i.e. Grief Rumination (Fig. 2 for the path diagram). Inspection of the fit indices (see Table 2) indicated that model A showed a marginally better fit than model B; this is also borne out by the comparative fit indices with the exception of the parsimony-adjusted fit index (PCFI), which favours the second model. For both models, all regression weights were significant (*p* < .001).

**Table 2:** Fit indices for the models for the UGRS tested in the confirmatory factor analysis

Model	χ^2^	df	*p*	χ^2^/df	RMSEA [90% CI]	SRMR	CFI	PCFI	AIC
A	119.72	80	.003	1.50	.056 [.034; .076]	.054	.969	.738	229.72
B	132.31	85	<.001	1.59	.059 [.039; .078]	.064	.963	.780	232.31

Legend: Model A: five intercorrelated factors; Model B: five factors with one higher-order factor. *RMSEA* Root mean square error of approximation, *SRMR* Standardised root mean residual, *CFI* Comparative fit index, *PCFI* Parsimony-adjusted CFI, *AIC* Akaike information criterion

### Validity

The UGRS sum score demonstrated significant correlations with both brooding and reflection. However, the correlation of the UGRS with brooding (*r*_UGRS x Brooding_ = .56, *p* < .001) was significantly higher than with reflection (*r*_UGRS x reflection_ = .30, *p* < .001) as indicated by the Fisher z-test [z(157) = 2.82, *p* < .005]. Interestingly though, the subscales showed varying associations with the rumination sub-facets brooding and reflection. ‘Injustice’ and ‘Counterfactuals’ were associated with brooding but not reflection, while all other subscales demonstrated significant correlations with both rumination sub-facets (see Table 3).

**Table 3:** Correlations of the UGRS and its subscales with measures of grief, depression, anxiety and rumination

	ICG	HADS-Anxiety	HADS-Depression	Brooding	Reflection
UGRS Sum Score	.76***	.51***	.48***	.56***	.30***
Meaning	.54***	.38***	.39***	.24**	.19*
Relationships	.61***	.47***	.42***	.38***	.28***
Counterfactuals	.52***	.38***	.32***	.42***	.12
Injustice	.59***	.30***	.30***	.57***	.12
Reactions	.51***	.35***	.36***	.32***	.43***

Legend: * *p* < .05; ** *p* < .01; *** *p* < .001; Bonferroni-corrected threshold: *p* < .0013 (all *p* with *** are significant after Bonferroni correction). *HADS* Hosptial Anxiety and Depression Scale, *ICG* Inventory of Complicated Grief

The UGRS sum score showed high correlations with symptoms of complicated grief symptoms as measured by the ICG. Correlations between the UGRS and indicators of anxiety and depression (HADS) were also significant. Using Fisher z-tests to compare the size of the coefficient *r*_UGRS x ICG_ with that of the coefficient *r*_UGRS x HADS-anxiety_ [z(157) = 3.80, *p* < .001] and *r*_UGRS x HADS-depression_ [z(157) = 4.13, *p* < .001], demonstrated that the latter two were significantly smaller. Considering the UGRS subscales, their correlation pattern with complicated grief, anxiety and depression generally followed the sum score’s pattern.

The hierarchical multiple regression on symptoms of complicated grief investigated the concurrent validity of the UGRS (see Table 4 for detailed results). It demonstrated in the first step that neither age, gender nor time since loss were significant predictors for ICG scores in this sample [*F*(3,114) = 1.452; *p* = .232, *adj.R*^*2*^ = .011]. When entered in the next step, both symptoms of anxiety and depression contributed significantly to the prediction of ICG scores [*F*(5,112) = 29.683; *p* < .001; *adj.R*^*2*^ = .551]. In the third model, brooding but not reflection explained a significant additional amount of variance [*F*(7,110) = 24.817; *p* < .001; *adj.R*^*2*^ = .588]. UGRS scores were included in the model in the final step and improved the prediction of ICG scores significantly [*F*(8,109) = 38.690; *p* < .001; *adj.R*^*2*^ = .720]. In this last model, regression coefficients for anxious and depressive symptoms remained significant predictors, while brooding lost its previous significance. This model explained 72% of variance in ICG scores.

**Table 4:** Results of the multiple hierarchical regression analysis

	B	SE B	β	ΔR^2^
Step 1 (df = 3, 114)				.037
Constant	25.078	9.470	–	
Age	−0.024	0.117	−.020	
Gender	5.283	3.429	.145	
Time since loss	3.304	2.800	.109	
Step 2 (df = 2, 112)				.533***
Constant	−11.229	7.096	–	
Age	−0.051	0.080	−.041	
Gender	2.497	2.377	.068	
Time since loss	−0.977	1.923	−.032	
HADS-depression	1.487	0.219	.504***	
HADS-anxiety	1.119	0.244	.348***	
Step 3 (df = 2, 110)				.042**
Constant	−15.458	7.067	–	
Age	0.011	0.078	.009	
Gender	1.586	2.315	.043	
Time since loss	−1.639	1.866	−.054	
HADS-depression	1.446	0.216	.490***	
HADS-anxiety	0.898	0.243	.279***	
Brooding	1.012	0.298	.253**	
Reflection	−0.242	0.325	−.052	
Step 3 (df = 1, 109)				.127***
Constant	−17.326	5.824	–	
Age	−0.014	0.065	−.011	
Gender	1.496	1.906	.041	
Time since loss	−2.436	1.540	−.081	
HADS-depression	1.013	0.187	.344***	
HADS-anxiety	0.561	0.205	.174**	
Brooding	0.169	0.271	−.042	
Reflection	−0.175	0.267	−.038	
UGRS	0.511	0.070	.503***	

Legend: Results of the multiple hierarchical regression with forced blockwise entry. Criterion: complicated grief symptoms (ICG Score). Demographic characteristics and time since loss entered in the first block, depressive and anxiety symptoms in the second, brooding and reflection in the third, and grief-related rumination (UGRS Score) in the last block. ** *p* < .01, *** *p* < .001. Only participants were included whose loss occurred more than six months ago. Gender was dummy-coded; Time since loss was dummy-coded with 0 indicating a loss 12 to 36 months ago and 1 indicating a loss 6 to 12 months ago

In order to investigate the discriminant validity of the UGRS, two groups of candidates for possible diagnosis of complicated grief were established based on the ICG cut-off score and different timing criteria (> 6 months for ICD-11; > 12 months for DSM 5). The UGRS sum score and its subscales differed significantly between the respective candidate groups for a diagnosis of complicated grief and groups of participants with the same time since loss but whose grief symptoms remained below the cut-off, with higher UGRS scores in the candidate groups (see Tables 5 and 6).

**Table 5:** Candidates and Non-Candidates for complicated grief (time since loss > 12 months)

	CG candidates (*n* = 61)		Non-CG candidates (*n* = 27)		*t*	*df*	Cohen’s *d*
	Mean	SD	Mean	SD			
UGRS Sum Score	50.50	12.39	33.52	7.41	7.96***	78.239	1.66
Meaning	12.87	2.75	10.04	3.04	4.31***	86	0.98
Relationships	8.72	3.58	5.33	2.09	5.55***	79.266	1.16
Counterfactuals	9.03	4.00	5.78	3.15	4.10***	62.471	0.90
Injustice	9.48	4.42	4.85	2.20	6.54***	84.773	1.32
Reactions	10.40	3.30	7.52	2.89	3.92***	86	0.93

Legend: *CG* complicated grief. Independent *t*-tests comparing candidates for complicated grief (ICG > 25) compared to persons below the cut-off (ICG ≤ 25), both with time since loss more than 12 months. If Levene’s test indicated that variances were unequal, the Welch test is reported (and the degrees of freedom adjusted accordingly). *** *p* < .001

**Table 6:** Candidates and Non-Candidates for complicated grief (time since loss > 6 months)

	CG candidates (*n* = 89)		Non-CG candidates (*n* = 32)		*t*	*df*	Cohen’s *d*
	Mean	SD	Mean	SD			
UGRS Sum Score	51.34	11.82	34.88	8.45	8.44***	76.566	1.60
Meaning	13.18	2.47	10.25	2.92	5.50***	120	1.09
Relationships	8.93	3.31	5.56	2.49	5.98***	72.635	1.15
Counterfactuals	9.01	3.88	5.84	3.34	4.41***	62.837	0.87
Injustice	9.72	4.39	5.66	3.17	5.60***	75.562	1.06
Reactions	10.51	3.13	7.56	2.77	4.70***	119	1.00

Legend: *CG* Complicated grief. Independent *t*-test comparing candidates for complicated grief (ICG > 25) to persons below the cut-off (ICG ≤ 25), both with time since loss more than 6 months. If Levene’s test indicated that variances were unequal, the Welch test is reported (and the degrees of freedom adjusted accordingly). *** *p* < .001

## Discussion

This is the first study to present and validate a German version of the UGRS. In a sample of recently bereaved participants, the German UGRS was shown to have an identical factor structure to the original UGRS and the UGRS and its subscales demonstrated very good item properties, internal consistency and convergent, divergent, concurrent and discriminant validity.

The reliability of the UGRS in this study is high and comparable to those found for the English and Dutch versions. The internal consistencies of the subscales are – considering their extreme brevity – also very good. All item difficulties are in the medium range, which is recommended for maximum discriminatory power. Overall, the item-whole correlations with the total scale were high, with the exception of item 10, which was in the medium range.

Concerning the factorial structure, our analyses mirror the result of the original analyses in English and Dutch samples. They demonstrated that, even though the lower-order model with five correlated factors provided the best fit, a higher-order model with Grief Rumination as a second-order latent construct was only marginally outperformed [19]. Importantly, the fit indices of both competing models differed even less in our sample compared to the original analyses. Both absolute and comparative fit indices award a very small advantage to the lower-order model, albeit only marginally. Parsimony is an important aspect in evaluating model fit. Of note, the parsimony-adjusted fit index PCFI favours the hierarchical model. This argument for conceptualising the UGRS as containing one higher-order factor dovetails with the theoretical viewpoint, because grief rumination was intended as a unidimensional construct in which the subscales each represent a recurrent theme of repetitive thinking. Naturally, the themes will vary among bereaved people and not all themes will occur equally often in bereaved persons. However, the latent process of grief-specific rumination is thought to represent the unifying construct underlying these themes. Given the only marginal superiority of a lower-order model in the confirmatory factor analysis results of the English and Dutch UGRS [19] and our own data, it seems preferable to conceptualise the UGRS sum score as an indicator of grief-specific rumination.

The validity of the UGRS was supported further in our sample. Importantly, supporting the convergent and divergent validity of the UGRS, grief rumination was more closely associated with maladaptive types of ruminative thought (i.e. brooding) than more adaptive types (i.e. reflection). This replicates previous findings [19] even to the point that the UGRS subscales Injustice and Counterfactuals demonstrated no significant association with reflection. Both the UGRS sum score and all subscales showed high correlations with complicated grief. This concurs with theoretical accounts that view rumination as a mechanism which perpetuates complicated grief [8]. Associations between the UGRS and symptoms of anxiety and depression, which are often present concurrently in persons who suffer from complicated grief [43], were significant, yet the level of the associations also differed significantly: grief rumination was more closely associated with disabling grief than with symptoms of depression or anxiety, providing a first indication of the UGRS’s discriminant validity. The regression analysis additionally demonstrated that the association between UGRS scores and symptoms of complicated grief holds even when simultaneously considering demographic and loss-related variables, depressive or anxiety symptoms, and other facets of rumination. The proportion of variance explained was high. Of note, UGRS scores explained incremental variance in ICG scores, over and above the aforementioned constructs. This speaks for the importance of grief-specific rumination as an independent construct.

Lastly, we investigated whether UGRS scores differed between participants with and without an increased likelihood of receiving a diagnosis of complicated grief. To this end, we classified participants according to the established symptom level cut-off in the ICG as candidates or non-candidates for complicated grief, applying different time criteria. The loss had to have happened at least six months ago (time criterion ICD-11) or twelve months ago (time criterion DSM 5). Irrespective of the time criterion, the UGRS scores were elevated strongly (Cohen’s *d* = 1.66 and *d* = 1.60) in both candidate groups when compared with the group with less likelihood of receiving a diagnosis of complicated grief. This speaks for the discriminant validity of the UGRS.

Several limitations must be borne in mind when interpreting the results. All data are based on online self-reporting and are cross-sectional in nature. The sample consisted predominantly of conjugally bereaved females, which mirrors the over-representation of this subgroup in most grief research [44], but is not representative of the general bereaved population. While we have no reasons to assume that the associations under investigation are different for lower-educated people and men, future studies on rumination following loss should aim to recruit more representative samples. The findings refer to a convenience sample of bereaved persons, which allows comparison with previous UGRS psychometric research, but they require replication in a clinical sample of patients who suffer from complicated grief in order to ascertain that the associations remain stable across the complete range of disabling grief symptoms. Complicated grief is a diagnosis that is undergoing (re-) conceptualisations with the revisions of the classification systems. Even though the ICG is one of the best established and most-used instruments for assessing disabling grief symptoms, it is unclear to what extent the ICG and its established cut-offs concur with the diagnostic criteria of Persistent Complex Bereavement Disorder (DSM 5) or Prolonged Grief Disorder (ICD-11), which limits the generalisability of our results. Additionally, information on grief symptom levels in our sample was based on self-report: no clinical diagnosis can be made based solely on this information. Therefore, we have referred to our diagnostic categories based on the ICG cautiously as ‘candidates for complicated grief’ in order to account for this diagnostic uncertainty.

## Conclusion

Further knowledge about the role of grief rumination may benefit the optimal treatment of patients who suffer from complicated grief: if grief rumination is an avoidance strategy (RAH [10]), psychotherapy should be tailored to address it, for instance by using exposure therapy. First evaluations of grief treatments that include interventions specifically targeting grief-related rumination have shown promising results concerning complicated grief [45, 46]. If, however, rumination is a confrontation strategy (RST [11]), distraction may be more beneficial. Value-oriented behavioural activation could represent one therapeutic strategy to achieve this goal; its clinical usefulness for grief treatment has already been demonstrated [46–48]. Based on these seemingly contradictory accounts, a recent review concludes that the two frameworks may be complementary and that there may be more than one way to effectively address grief rumination [13]. Thus, future research is needed to establish (1) whether grief rumination is an aetiological or a maintaining factor in complicated grief; (2) which theoretical framework best explains its role in adjustment to bereavement; and (3) whether reductions in grief rumination levels mediate the effectiveness of interventions for severely distressed bereaved persons. Establishing validated instruments to assess malleable cognitive constructs relevant to complicated grief, such as grief rumination will likely contribute to this goal.

## Additional file

Additional file 1: German Version of the Utrecht Grief Rumination Scale. (PDF 60 kb)

## Abbreviations

CG: Complicated grief
DSM 5: Diagnostic and Statistical Manual for Mental Disorders, 5th edition
HADS: Hospital Anxiety and Depression Scale
ICD-11: International Classification of Diseases, 11th edition
ICG: Inventory of Complicated Grief
PCBD: Persistent Complex Bereavement Disorder
PGD: Prolonged Grief Disorder
RAH: Rumination as Avoidance Hypothesis
RSQ 10D: Response Styles Questionnaire 10D
RST: Response Styles Theory
UGRS: Utrecht Grief Rumination Scale
