# Supplementary material for: Assessment of grief-related rumination: validation of the German version of the Utrecht Grief Rumination Scale (UGRS)
Source: BMC Psychiatry. 2018 Feb 9;18:43. doi: 10.1186/s12888-018-1630-1 (PMC5807860; doi:10.1186/s12888-018-1630-1)
Supplement: Supplementary file 1 — German Version of the Utrecht Grief Rumination Scale. (PDF 60 kb) [file 12888_2018_1630_MOESM1_ESM.pdf]

Utrecht Grief Rumination Scale – Deutsch (UGRS-D)

Gedanken über den Verlust

Nach dem Tod einer geliebten Person denken Menschen oft (an) ganz verschiedene Dinge. In den folgenden Fragen möchten wir gerne wissen, wie oft Sie in dem vergangenen Monat über die folgenden Dinge nachgedacht haben.

**Wie häufig im vergangenen Monat haben Sie…**	**Nie**	**Manch-mal**	**Regel-mäßig**	**Oft**	**Sehr oft**
1. …über die Folgen nachgedacht, die ihr/ sein Tod für Sie hat.	①	②	③	④	⑤
2. …genau überlegt, welche persönliche Bedeutung dieser Verlust für Sie hat.	①	②	③	④	⑤
3. …sich gefragt, ob Sie die richtige Unterstützung von Familienmitgliedern erhalten.	①	②	③	④	⑤
4. …genau überlegt, ob Sie ihren/ seinen Tod hätten verhindern können.	①	②	③	④	⑤
5. …sich gefragt, womit Sie diesen Verlust verdient haben.	①	②	③	④	⑤
6. …versucht, Ihre Gefühle bezüglich dieses Verlusts genau zu ergründen	①	②	③	④	⑤
7. …sich gefragt, ob Ihre Reaktionen auf diesen Verlust normal sind.	①	②	③	④	⑤
8. …sich gefragt, ob ihr/ sein Tod hätte verhindert werden können, wenn die Umstände anders gewesen wären.	①	②	③	④	⑤
9. …sich gefragt, ob Sie angemessene Unterstützung von Freunden und Bekannten erhalten.	①	②	③	④	⑤
10. …sich gefragt, ob ihr/ sein Tod hätte verhindert werden können, wenn andere Menschen anders gehandelt hätten.	①	②	③	④	⑤
11. …sich gefragt, warum dies Ihnen und nicht jemand anderem passieren musste.	①	②	③	④	⑤
12. …über die Ungerechtigkeit dieses Verlustes nachgedacht.	①	②	③	④	⑤
13. …versucht, Ihre Gefühle bezüglich des Verlusts zu verstehen.	①	②	③	④	⑤
14. …nachgedacht, was Sie sich für eine Reaktion von Anderen auf Ihren Verlust wünschen	①	②	③	④	⑤
15. …nachgedacht, wie sich Ihr Leben durch ihren/seinen Tod verändert hat.	①	②	③	④	⑤

©Doering, Barke, Friehs & Eisma, 2018

Der Gesamtwert der UGRS wird durch Summierung der Einzelitems gebildet. Subskalenwerte berechnen sich durch Summierung der zugehörigen Items:
Bedeutung des Verlustes: 1, 2, 15
Soziale Unterstützung: 3, 9, 14
Kontrafaktisches Denken: 4, 8, 10
Ungerechtigkeit: 5, 11, 12
Trauerreaktionen: 6, 7, 13

Bitte zitieren Sie bei Verwendung der deutschen Übersetzung als Referenz den entsprechenden Artikel in BMC Psychiatry

©Doering, Barke, Friehs & Eisma, 2018
